# Context and repetition in word learning

**DOI:** 10.3389/fpsyg.2013.00149

**Published:** 2013-04-09

**Authors:** Jessica S. Horst

**Affiliations:** School of Psychology, University of SussexBrighton, UK

**Keywords:** word learning, fast mapping, shared storybook reading, contextual repetition, language acquisition

## Abstract

Young children learn words from a variety of situations, including shared storybook reading. A recent study by Horst et al. (2011a) demonstrates that children learned more new words during shared storybook reading if they were read the same stories repeatedly than if they were read different stories that had the same number of target words. The current paper reviews this study and further examines the effect of contextual repetition on children's word learning in both shared storybook reading and other situations, including fast mapping by mutual exclusivity. The studies reviewed here suggest that the same cognitive mechanisms support word learning in a variety of situations. Both practical considerations for experimental design and directions for future research are discussed.

Across all **word learning** situations, each “known” word first begins as a novel word (Horst et al., 2006; see also Gathercole, 2006). The first time a word is encountered, the child (or other learner) is provided with an opportunity to store some information about that word, for example, how it sounded, who said it, which possible referents were present, etc. Although some learning does occur during **fast mapping** when words are initially encountered (Horst et al., 2006; Carey, 2010), one encounter seldom provides sufficient experience to support robust word learning (Horst and Samuelson, 2008; Mather and Plunkett, 2009). Importantly, then, as the word is repeatedly encountered, additional opportunities to store relevant information are presented, facilitating the creation of a more robust representation (see also, Yu and Smith, 2007; Horst and Samuelson, 2008; McMurray et al., 2012). Over time, the word will become increasingly familiar as the child learns some of the statistical regularities of how the word is used. Eventually, the child is able to reliably detect the word's referent, even after delays and in a variety of contexts, and ultimately to produce the word himself—at these points we might refer to the word as a “known word.” Thus, novel and known words exist on a continuum of novelty to familiarity. Each word begins as a novel word. Through repeated exposures across a variety of contexts any word has the potential to become a known word. Moreover, the phrase “novel word” (and also “novel object”) is really shorthand for academics to differentiate between names of varying degrees of familiarity.

One common way in which young children encounter new words is via **shared storybook reading** (Sénéchal, 1997). As early as 8 months of age, children begin learning words that frequently occur in stories read to them (Jusczyk and Hohne, 1997). Before age six, up to 80% of children are read a story each day (Rideout et al., 2003). As any parent can attest, it is not uncommon for young children to ask for a book (or video) to be repeated (Sulzby, 1985; Crawley et al., 1999). Further, repetition can actually increase enjoyment from videos and stories (Crawley et al., 1999; Leavitt and Christenfeld, 2011). Importantly, repeatedly being read the same storybook facilitates word learning (Sénéchal, 1997; Horst et al., 2011a; McLeod and McDade, 2011; Wilkinson and Houston-Price, in press). Moreover, the number of times parents read to their children and the number of trips to the library predict both children's receptive and expressive vocabulary sizes (Arterberry et al., 2007). Thus, shared storybook reading clearly plays an important role in early word learning.

Recently, Horst et al. (2011a) demonstrated that 3-year-old children learned more novel words from shared storybook reading when the same three storybooks were read repeatedly, than when nine different storybooks were read. All children performed well on the initial test immediately following the shared storybook reading, however, only children who had heard the same stories repeatedly retained the word-object associations when tested 1 week later. That is, only for these children did the novel words become known words. The authors explain their results in terms of the benefit of **contextual repetition**. Through repeated exposures to the same storybook texts and illustrations children are able to form a robust representation of a new word because such contextual repetition helps lower the attentional demands of word learning. Focusing on the cognitive processes at work during shared storybook reading helps us illuminate the domain-general learning mechanisms that support such word learning, effectively bridging the gap between our understanding of how children learn in naturalistic settings, such as when a parent reads a storybook, to more artificial settings, such as fast mapping by mutual exclusivity experiments. Moreover, demonstrating that the same cognitive mechanisms support word learning across situations not only informs our understanding of children's word learning via shared storybook reading or word learning via fast mapping, but also our insight into **language acquisition** more generally.

## Context and repetition in word learning via storybooks

Several studies have demonstrated an advantage for repeatedly reading storybooks to young children (for a review see, Biemiller and Boote, 2006). For example, Sénéchal (1997) read 3-and 4-year-old children a story either once or three times. Children learned significantly more words in the repeated reading conditions than in the single reading condition. Similarly, Biemiller and Boote (2006) demonstrated that young school children learned more words after hearing stories read four times than after hearing them only read twice. When stories are read only twice, children learned more words if the words had occurred twice in the story (four exposures) than if they had occurred once in the story (two exposures, Robbins and Ehri, 1994). In a recent study by McLeod and McDade (2011), 3- and 4-year-old children shared either a single reading of a storybook that included each target word three times (three total exposures) or three readings of a storybook that included each target word once (also three total exposures). Overall, children learned more words when the same story was read repeatedly than when a single story was read, despite having the same number of total exposures.

However, the amount of time children in the different groups spent engaged in shared storybook reading was not always the same in these previous studies. For example, children randomly assigned to control groups sometimes only hear a single storybook once (Sénéchal, 1997; McLeod and McDade, 2011) and sometimes do not hear any storybooks (see, Lonigan et al., 2008, for a review). In addition, many previous studies have also included up to ten (e.g., Robbins and Ehri, 1994) or even 20 (e.g., Elley, 1989) target words, although young children between ages 1 and 6 can apparently only learn on average three words per day (Bion et al., 2013), which likely explains why the level of word learning in such studies rarely exceeds 20% (see Biemiller and Boote, 2006, for a review).

Recently, Horst et al. (2011a) controlled for these experimental design issues. Specifically, they provided all children with the same amount of overall story exposure. In addition, they only introduced two novel words per story (six words over the course of the study) because shared storybook reading studies have consistently reported that pre-school-aged children only learn on average up to two words per day (see Biemiller and Boote, 2006). School-aged children may be able to learn up to five words via repeated storybook reading (Wilkinson and Houston-Price, in press). Likewise, Horst et al. (2011a) only introduced novel nouns because children do not learn verbs and adjectives as well as nouns via storybooks (see, Ard and Beverly, 2004 for a review). The authors used purpose-written storybooks rather than commercially available storybooks to ensure that stories were equally interesting for both groups, similar in length and, importantly, that each target word occurred the same number of times, which is known to be an issue when using commercially available storybooks for research (Robbins and Ehri, 1994). The use of purpose-written storybooks also allowed Horst et al., to use novel words to certify that any learning children demonstrated was due to the experimental manipulation and not *a priori* knowledge (for a similar argument see, Bornstein and Mash, 2010).

**Figure 1:**
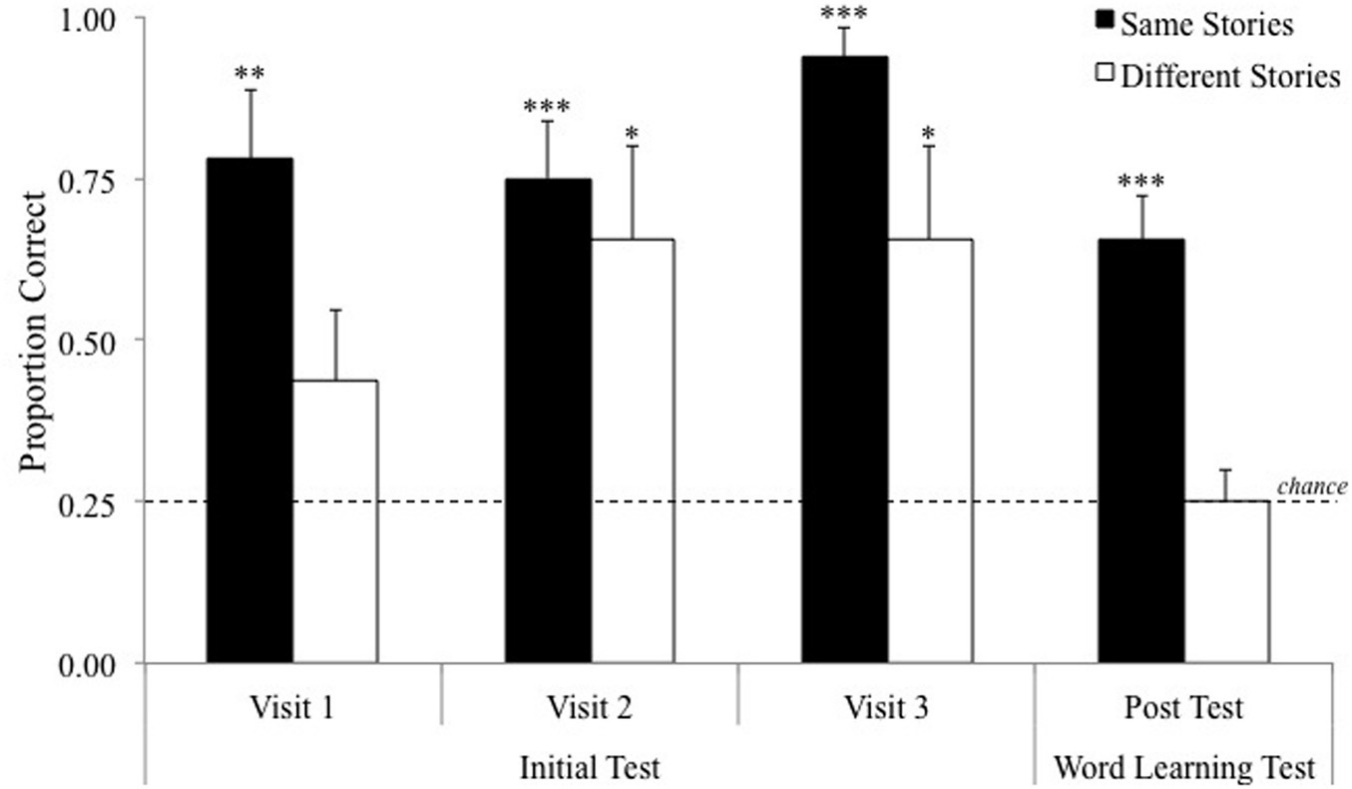
**Results from Horst et al. (2011a) at a glance.** Dotted line represents chance (0.25). Error bars represent one standard error. ^***^p < 0.001, ^**^p < 0.01, and ^*^p < 0.05. All *p*s are two-tailed.

Horst et al. (2011a) created nine storybooks that each depicted two novel objects. For example, *The Very Naughty Puppy, Nosy Rosie at the Restaurant* and *Rosie's Bad Baking Day* each depicted both the *sprock* and the *tannin*. Each novel object was depicted and named exactly four times in each story. In one group, children were read three different stories on each of 3 days (i.e., nine story exposures to nine different stories). In another group, children were read the same story three times and a different story on each of 3 days (i.e., nine story exposures to three different stories). Importantly, all children were exposed to the target words the same number of times (12 exposures per target word). After each shared storybook reading episode, children were tested on their immediate recall for that day's target words. All children performed well on this test, however, children who had heard the same stories repeatedly recalled significantly more words (see Figure 1). Importantly, at the end of the study, children were tested on their retention for the words from days 1 and 2, which they had heard 3–6 days earlier but had not heard since. Only children who had heard the same stories repeated retained the word-object associations. Moreover, children who had heard different stories performed at chance levels.

Horst et al. (2011a) argue that hearing the same stories repeatedly facilitated word learning due to a contextual cueing effect. Contextual cueing is a form of implicit and incidental learning, which refers to an advantage in visual cognition tasks when contexts (backdrops) are repeated over learning (for a review, see Chun, 2000). Originally coined to explain the facilitative effects of visual search when spatial locations of distractors are repeated (Chun and Jiang, 1998), contextual cueing and related effects have been observed with several types of visual stimuli, for example landscape paintings (Kornell et al., 2010). Specifically, contextual cueing refers to how repeated contexts guide (or cue) attention to a to-be-learned target (Chun, 2000). The idea underlying such contextual repetition is that because the visual world is highly structured, redundant and predictable, context facilitates recognition of objects within a scene (Meints et al., 2004). Therefore, contextual repetition serves to both reduce complexity and increase predictability. Children benefit from predictability, which may also explain their attraction to highly structured and predictable games (e.g., duck, duck, gray duck), songs (e.g., Old McDonald) and books (e.g., Seuss, 1960).

As applied to shared storybook reading, the idea is that each time the child hears a story, more attention can be devoted to the target and less attention to the plot and other irrelevant-for-word-learning aspects. For example, a child's first encounter with a storybook may require high attentional demands as the child focuses on the overall plot, the setting, who the characters are, etc. Because these aspects will be somewhat familiar when the story is read again, attentional demands will be a little lower, allowing the child to focus on other aspects of the story (see also, Leavitt and Christenfeld, 2011). That is, repetition may help draw children's attention from highly salient aspects of the storybook to smaller details including new words (see also, Crawley et al., 1999; Mares, 2006; Perrachione et al., 2011, for similar arguments in other domains). This contextual repetition account also explains McLeod and McDade's (2011) findings. Recall, in that study all children encountered the target words exactly three times. However, the children whose second and third encounters were in the context of a repeatedly read storybook learned the words, whereas children whose second and third encounters were in the context of a single reading of a single storybook did not.

Across several studies, children have been read the same storybooks immediately again, however, in everyday life, children are sometimes read only one story per shared storybook reading episode. When children do hear the same storybook repeatedly read, this repetition may occur after a substantial delay, such as the next day. If the contextual repetition explanation is correct, then we should see the same effect when children encounter the same stories over a longer time scale such as across several days. In a recent replication of the target study, two groups of children were again read the same stories or different stories over the course of 1 week (Williams et al., 2011). However, each day all children heard three different stories each containing two target words (six words total). For example, 1 day a child heard *The Very Naughty Puppy* (*sprock, tannin*), *Mischief at the Toyshop* (*manu, zorch*) and *The Surprisingly Good Bad Day* (*coodle, gaz*). On the second and third days, children in the different stories conditions heard new stories (i.e., nine story exposures to nine different stories) and children in the same stories conditions heard the same stories as on the first day (i.e., nine story exposures to three different stories). Again, children benefitted from repeatedly reading the same stories. Specifically, children who heard different stories recalled 44% of the novel names on the final day and children who heard the same stories repeatedly recalled 60% of the novel words on the final day. Note this rate is lower than in the target study because children were exposed to six different words each day (as opposed to two words per day in the target study). A similar contextual repetition effect has also been observed in the classroom (Wilkinson and Houston-Price, in press). Teachers read their classes one book each week for 3 weeks. Six- and seven-year-old children learned more words when they were read the same book repeatedly than if they were read three different books, although all children heard each word exactly nine times.

Overall, these studies demonstrate a clear advantage for repeated readings to facilitate word learning via shared storybook reading. The contextual repetition that occurs when children hear a story read repeatedly supports word learning by lowering the attentional demands of the word learning task. Although shared storybook reading is one important way in which children learn words it is not the only way, thus, the next question is whether contextual repetition facilitates word learning in general. In particular, we can investigate whether the same general cognitive mechanisms are responsible for word learning more generally by investigating a distilled, stripped-down task such as learning via fast mapping by mutual exclusivity. If the same cognitive mechanisms support word learning in a variety of situations we should see similar results in both shared storybook reading and fast mapping studies.

## Context and repetition in other word learning situations

Recently, McMurray et al. (2012) have argued that word learning is a slow process via gradual **associative learning**. Over time, across multiple encounters, children are able to learn the association between the word and referent (Smith and Yu, 2008; McMurray et al., 2012). For example, a child might hear the word “rake” in the presence of a rake, a lawnmower and a wheel barrow. Or a child might hear the word “rake” while looking at a page in *The Cat in the Hat* (Seuss, 1957). If the same domain-general cognitive mechanisms support word learning in a variety of situations, then those that support word learning via shared storybook reading should be the same as those that support word learning while naturally playing a social game with a parent or word learning via mutual exclusivity style fast mapping trials in a laboratory experiment. That is, the same cognitive mechanisms that help a child determine “rake” refers to a rake in their backyard should be the same as those that help a child determine “rake” refers to a specific object on a storybook page. Further, general cognitive mechanisms such as focusing attention and learning statistical regularities should help across a variety of word learning situations. Consequently, we should expect similar manipulations to lead to similar results across a variety of word learning situations.

In cases where children appear to quickly learn a new word in the context of other items children need to attend to the correct items at the correct time (Axelsson et al., 2012). For example, they need to attend to the lawnmower and wheel barrow to rule them out as the referent of “rake” but they also need to attend to the rake to encode something about it to facilitate learning the “rake”-rake word-object association (see also, Horst and Samuelson, 2008; Mather, 2013). This is quite similar to learning words via shared storybook reading where children need to attend to and encode the word-object association in the context of an illustration in a storybook and the prose. In both situations, word learning appears to require both attention to the targets and decreasing attention/avoiding non-targets.

Increasing attention to targets can be accomplished in a variety of different ways including repetition (Mather and Plunkett, 2009), **ostensive naming** (Axelsson et al., 2012), gesture (McGregor et al., 2009), social-pragmatic cues (Moore et al., 1999) etc. Similarly, decreasing attention to non-targets can also be accomplished in a variety of different ways including keeping them nameless (Jaswal and Markman, 2001), covering them (Axelsson et al., 2012), decreasing the number of them (Horst et al., 2010) and removing them from view (Dollaghan, 1985). Several studies of child word learning have, in fact, used these methods to simultaneously increase children's attention to the target(s) and decrease their attention to the non-targets (Woodward et al., 1994; Akhtar et al., 1996; Horst and Samuelson, 2008).

**Figure 2:**
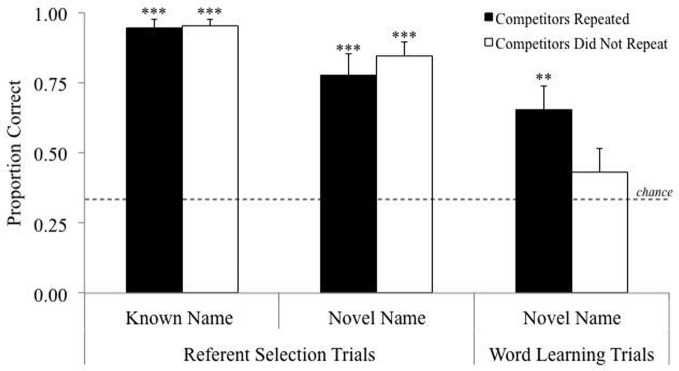
**Results from repeated competitors experiment.** Dotted line represents chance (0.33). Error bars represent one standard error. ^***^p < 0.001, ^**^p < 0.01. All *p*s are two-tailed.

Contextual repetition appears to draw children's attention to the target words and away from other aspects of the storybook context. Whether contextual repetition facilitates word learning via fast mapping was recently tested using a touch-screen computer paradigm (Horst, 2011). Children were presented with two known **competitors** and one novel target object on each **referent selection** trial. Each novel target was presented three times across referent selection trials. For one group of children the competitors were always different across trials for a given novel target (for example, the clacker was presented once with the frog and ball, once with the cow and train and once with the elephant and cup). For another group of children the competitors were always the same across trials for a given novel target (for example, the clacker was always presented with the frog and ball). Importantly, across trials children saw each object the same number of times. All children were given the same test trials with only the novel targets (cf. retention trials). As can be seen in Figure 2, children in both groups did equally well on the initial referent selection task. However, only children who were given contextual repetition (i.e., repeated competitors) demonstrated word learning at test. Because children in both groups had identical amounts of exposure to the novel targets and had seen the non-targets the same number of times, we can confidently conclude that this effect is due to whether or not children saw the same competitors each time they encountered the novel target, which was the only difference between the two groups. Importantly, these findings suggest the same cognitive mechanisms facilitate word learning in both shared storybook reading and fast mapping situations.

Other language studies have also found important effects of contextual repetition. For example, Mather and Plunkett (2009) demonstrated toddlers can recall the context in which they had seen a novel target. They presented 22.5-month-old children with both a known object (e.g., car) and a novel object (e.g., compass) and directed children to look at either the known or novel object. Later in the experiment they presented the same trials again (e.g., car and compass). When the context repeated, toddlers looked significantly longer at the target *before* they heard the target name (for both known and novel name trials). That is, when the context (target paired with competitor) was repeated, toddlers were able to focus their attention onto the target before they were instructed to do so. This finding reveals that young children can retain some information about the context in which objects are presented, remember this contextual information during the intervening trials and, if repeated, such contextual cues can help guide attention.

However, in general, word learning occurs via gradual, associative learning (McMurray et al., 2012) and when children learn a target word-object association in a context (e.g., “rake”-rake in the context of a lawnmower and wheel barrow) they are also preventing and pruning spurious associations (e.g., “rake”-lawnmower and “rake”-wheel barrow). This suggests, then, that more variable contexts may facilitate word learning. Indeed, language acquisition research shows an advantage for increased variability during learning (e.g., Gómez, 2002; Singh et al., 2008; Rost and McMurray, 2009; Perry et al., 2010; Thiessen, 2012). And yet the studies reviewed here demonstrate an advantage for decreased variability during learning (Horst et al., 2011a; McLeod and McDade, 2011; Wilkinson and Houston-Price, in press). Importantly, what is varied in these studies is not the same. Because word learning benefits from increasing attention to the target and decreasing attention to the competitors (Axelsson et al., 2012), manipulating either (or both simultaneously) may improve word learning. One possible explanation for these different findings, then, is that increasing variation to the target stimulus helps to *increase attention to the targets* while contextual repetition helps to *decrease attention to the not-to-be-learned items* (e.g., competitors/non-targets, background).

In general, studies that have found an advantage for variability have increased variation to the targets (e.g., Perry et al., 2010). In these studies the target is also typically occurring in a context with other to-be-learned items, such as other phonetic sounds (e.g., Rost and McMurray, 2009, 2010), and children may be unaware of which elements in the context are the to-be-learned targets (Apfelbaum et al., in press). In contrast, studies that have found an advantage for contextual repetition, have decreased variation to the competitors/non-targets and background (Horst et al., 2011a; McLeod and McDade, 2011; Wilkinson and Houston-Price, in press). It is also important to note that even in such studies children encountered each target word across multiple storybook pages that is, children encountered some variability across a small number of different contexts.

Thus, it is possible that there could be a “sweet spot” for variability: one requires enough contextual support and variability to encode a rich representation of the target, but not so much as to create such high attentional demands that too little information is encoded. Evidence in support of this notion exists in studies on fast mapping by mutual exclusivity. Specifically, children fail to retain recently fast-mapped targets encountered in the absence of any competitors (Zosh et al., 2013, i.e., no contextual support), but they do retain targets encountered among a small number of competitors (e.g., 1, Zosh et al., 2013; 2, Horst et al., 2010, i.e., low attentional demands) and again fail to retain targets encountered with yet more competitors (Horst et al., 2010, i.e., high attentional demands). It is also possible that the amount of variability and contextual repetition that is needed to support word learning changes over development as working memory capacity and attention spans increase. Consequently, variability may be less effective for novice learners (for reviews, see Wulf and Shea, 2002; Singh et al., 2008) and more desirable for proficient learners (Zosh et al., 2013). Future research is needed to further understand the complex interplay between variability at the target level and reduced variability at the non-target level (for a similar argument see, Apfelbaum et al., in press).

## Considerations for word learning and storybook research

Those who read stories to children have likely realized that children require more than a single exposure to a storybook to learn words in this situation (Sénéchal, 1997). Thus, far, we have reviewed how repetition and context facilitate word learning via shared storybook reading. There are, however, additional considerations for research and interventions using storybooks to promote vocabulary acquisition. Some of these considerations also apply to other word learning research as well.

First, the number of targets (i.e., new words) should be considered. Several studies have reported children learning approximately 3–4 new words during the course of 1 week via shared storybook reading—regardless of how many new words were introduced (e.g., Elley, 1989; Sénéchal and Cornell, 1993; Brett et al., 1996). Thus, the task may be too challenging if too many words are introduced at once. This could further decrease children's interest in the stories and their willingness to complete all test trials. Further, with too many targets, children typically exhibit only 20% accuracy (Biemiller and Boote, 2006). Such low learning rates could mask actual between group differences and, to obtain significance, require large sample sizes, which are expensive and time consuming. In addition, previous research demonstrates that all word types are not created equal and children learn nouns more easily from storybooks than other word types (e.g., verbs, adjectives, Robbins and Ehri, 1994; Ard and Beverly, 2004). If children do poorly, then, it is unclear whether this is due to the experimental manipulation or the types of words being introduced.

Second, the novelty of target objects must be considered. Previous research demonstrates that children have a endogenous bias to novelty (Horst et al., 2011b). Specifically, when presented with a novel word and no supportive information about the target referent (i.e., without known competitors), rather than responding randomly, children systematically link the novel word to the most novel object (see also, Mather and Plunkett, 2012). Importantly, this bias can be seen after as little as 2 min of exposure with previously novel objects. Thus, it is critical for experimental control that target novel objects are in fact novel and children do not come to the task with previous experience with them. Otherwise, it is unclear how much learning is due to the experimental manipulation or intervention or due to partial knowledge from previous experience (Bornstein and Mash, 2010).

Similarly, the novelty of target words must also be considered to ensure learning is due to the experimental manipulation or intervention and not *a priori* partial knowledge. Specifically, target words should be either completely novel pseudo-words (e.g., Wilkinson et al., 2003; Horst and Samuelson, 2008) or children should be pre-tested for word knowledge before participating (e.g., Thom and Sandhofer, 2009; Perry et al., 2010; Salas Poblete et al., under review). Both real words that are beyond children's current vocabulary levels (e.g., *attire, incline, trajectory*; Wilkinson and Houston-Price, in press) and “fake” pseudowords (e.g., *dack, sobe, tib*; McLeod and McDade, 2011) may be both appropriate for such research. Recall, from the child's perspective, they are both novel words because each word (real or created) begins as a novel word on the continuum of novelty to familiarity.

On a related note, the novelty of synonyms should be questioned. Precisely because novel and known words are on a continuum of familiarity, it may be difficult to pin point exactly when a novel word becomes a known word. Thus, adequate experimental control is especially important if we want to draw conclusions about how children learn new words from developmental research. Ard and Beverly (2004) argue that synonyms (e.g., *satchel* instead of *bag*) should not be used when testing children's word learning via shared storybook reading because learning a synonym only requires the child to extend a concept of a familiar word and associate a new word with that known concept (for a similar argument see, Sénéchal and Cornell, 1993). As such, the use of novel words for familiar referents may not accurately reflect how much the child did (or did not) learn from the experimental manipulation.

Third, the stories should be considered. Children have difficulty learning from books with plots and characters with whom they cannot easily identify (Elley, 1989). This makes intuitive sense. If children are especially unfamiliar with elements of a plot or characters, on repeated readings they may need to continue attending to these elements of the story to process them, which will defer attentional resources from attending to other elements of the story, such as new words (for a related argument, see Mares, 2006). This may also partly explain the poor performance in some previous studies.

Finally, the books themselves should be considered. Commercially available books are convenient and may be appropriate for some experimental research, particularly research focusing on dialogic reading techniques. However, as noted by several others (e.g., Cornell et al., 1988; Robbins and Ehri, 1994; Sénéchal et al., 1995), for research focusing on word learning, commercially available books may pose problems for experimental control, because books are different lengths, target words do not usually occur equally often throughout the story and different books may not be equally memorable. Each of these problems may lead to unintended differences between groups, creating confounds. Fortunately, these need not be problems for purpose-written storybooks, that is, storybooks designed for experimental use. Further, purpose-written storybooks can also benefit from recent research demonstrating that children learn more from books with realistic photographs and color drawings than from books with simple line drawings (Simcock and DeLoache, 2006; Ganea et al., 2009) and that children have a difficult time learning from books with manipulative features (e.g., pop-up books, Tare et al., 2010).

In addition, a rich literature demonstrates that dialogic techniques such as pointing to key items, elaborating on new concepts, asking questions, etc. facilitate word learning (for a review see, Blewitt et al., 2009). However, recent research has demonstrated successful word learning via shared storybook reading without the use of dialogic reading techniques (e.g., Horst et al., 2011a; McLeod and McDade, 2011). Depending on the focus of the research or intervention some or all of these elements of storybooks and novel words and objects (i.e., number of targets, novelty, synonyms, story design, book design) should be considered (see Table 1).

**Table 1:** **A summary of considerations for research and interventions for using shared storybook reading to improve vocabulary**.

	**Consideration**	**Optimal learning**	**For more information**
Words	Number of targets	2–5 words per story	Biemiller and Boote, 2006; Horst et al., 2011a; Wilkinson and Houston-Price, in press
	Word class	Nouns	Robbins and Ehri, 1994; Ard and Beverly, 2004
	Novelty	Novel words - or -Advanced vocabulary that is pretested	e.g., Horst et al., 2011a e.g., Wilkinson and Houston-Price, in press
		Avoid synonyms	Sénéchal and Cornell, 1993; Ard and Beverly, 2004
Illustrations	Illustration style	Color photographs	Simcock and DeLoache, 2006; Ganea et al., 2009
	Manipulative features	Avoid manipulative features	Tare et al., 2010
	Novelty	Novel objects (for nouns)	Bornstein and Mash, 2010; Horst et al., 2011b; Mather and Plunkett, 2012
Stories	Plots/characters	Relatable plots and characters	Elley, 1989
	Repetition	Three repeated readings	Sénéchal, 1997; Wilkinson and Houston-Price, in press
	Exposure	Same amount of shared storybook reading exposure between groups	Horst et al., 2011a; McLeod and McDade, 2011
	Books	Purpose-written storybooks (avoid commercially available books)	Robbins and Ehri, 1994; Wilkinson and Houston-Price, in press
	Reading Style	Dialogic techniques	Blewitt et al., 2009

Optimal Learning indicates the situations which have yielded the largest gains in word learning or have been recommended as best practice (e.g., avoiding synonyms). Depending on the research question being addressed the Optimal Learning scenarios may not be ideal (e.g., if a study seeks to investigate different word classes).

## Future directions

Horst et al. (2011a) argue that their findings are due to contextual repetition. This explanation is also consistent with findings from other studies using related methodologies (e.g., McLeod and McDade, 2011; Wilkinson and Houston-Price, in press). However, other alternative explanations for these findings remain. For example, because people enjoy hearing the same stories repeatedly (Leavitt and Christenfeld, 2011) it is possible that repeatedly hearing the same stories helped maintain children's attention in the same stories group. Another possibility is that because children benefit from predictable situations hearing a story re-read helped to guide their attention and alerted them to what was coming next. Alternatively, processing three different storybook plots may have overloaded children's attentional resources, making it especially challenging to notice and encode the novel words. Future research is needed to investigate these alternative explanations. We should bear in mind, however, that these explanations are not mutually exclusive and also do not preclude the role of contextual repetition. As in other areas of development, it is likely that children's learning was influenced by multiple factors working in tandem (Thelen and Smith, 1994).

Future research may also seek to explore when learning from different stories *does* occur. Clearly, in everyday life children are read the same stories repeatedly, however, many children are also read books only once (e.g., at storytime at the library or at a friend's house) or with a substantial lag between reading episodes (e.g., a child may hear *A Christmas Carol* (Dickens, 1844) only once annually). It is possible that children do learn words encountered across several different stories. The studies that have contrasted repeatedly reading the same stories or different stories have only read stories for 7–15 days (Horst et al., 2011a; McLeod and McDade, 2011; Wilkinson and Houston-Price, in press). It is possible that children do learn from different stories but that these studies have tested children too early in the process or before providing enough different stories (i.e., perhaps more than three different stories are needed). Alternatively, it is possible that what children learn from hearing the same story repeatedly or hearing different stories is not the same. Children who hear the same stories repeatedly may form narrower representations of new concepts whereas children who hear different stories may acquire a deeper understanding of the same concepts—but may require longer to do so. The existing studies have presented children with forced-choice comprehension trials using pictures. Children in the different stories conditions may have had an advantage over the children in the same stories conditions if they had been given different tests, such as production tasks (picture naming), extension trials (applying a new target word to a novel exemplar) or free recall and open-ended questions (e.g., “tell me what a *sprock* is used for.”). Thus, future research should also investigate how learning occurs via one-off shared storybook reading episodes and whether qualitative differences exist in what children learn depending on whether they learn via contextual repetition or variation.

The studies reviewed here support the view that the same cognitive mechanisms likely support word learning across various situations. For example, in both a shared storybook reading situation (Horst et al., 2011a; McLeod and McDade, 2011) and a mutual-exclusivity fast mapping touch-screen situation (Horst, 2011) contextual repetition facilitated word learning. Future research may seek to explore how other cognitive and socio-pragmatic mechanisms support word learning across a variety of situations, both naturalistic and experimental.

## Conclusions

During early childhood, engaging in shared storybook reading provides a common situation in which children are exposed to new words. Recent research in this area demonstrates that repeatedly reading the same stories is more effective for learning new words than reading several different stories (Horst et al., 2011a; McLeod and McDade, 2011). The goals of many parents engaged in shared storybook reading, however, are bonding and spending time together, not word learning *per se* (Audet et al., 2008). If the goals are bonding and spending time together, then whether they are reading storybooks best suited for building vocabularies may not be as important as reading storybooks that will invite conversations and engage children's imaginations. However, if the goal is word learning, then the idea of reading the same books repeatedly may be particularly encouraging for families who tend to borrow library books rather than buy books and who do not have large collections of storybooks at home, such as those from disadvantaged communities (Raikes et al., 2006). As such, continuing research in this area has important implications for parents, teachers and speech therapists.

Importantly, on the view that the cognitive mechanisms that support word learning via shared storybook reading are the same cognitive mechanisms that support word learning in other situations (e.g., fast mapping via mutual exclusivity, naturalistic play), insights from word learning from shared storybook reading can inform our understanding of word learning more generally. That is, what we learn with one method can be put to use in studies and interventions with other methods (e.g., that repetition facilitates learning that children can only learn a few words at once, etc.). Thus, the main studies reviewed here are not studies on shared storybook reading but rather studies on young children's word learning in which children encountered new words via storybooks.

### Conflict of interest statement

The author declares that the research was conducted in the absence of any commercial or financial relationships that could be construed as a potential conflict of interest.

## Key Concept

Word learning: The act of learning about a word (meaning, phonetic properties, etc.) such that it becomes a robust representation in one's vocabulary (for a review see, McMurray et al., 2012).
Fast mapping: An initial rough hypothesis of what a word might mean, not a full lexical representation (Carey, 2010).
Shared storybook reading: The act of two or more individuals simultaneously focusing their attention on the same storybook as it is being read aloud. For example, a parent reading an illustrated storybook to a child who is sitting next to her.
Contextual repetition: The repetition of a given situation. Examples include: seeing the same illustrations each time a given book is read, observing the same toys together (as with a farm set) or encountering the same object array repeatedly. Items need not be in the same spatial locations across encounters.
Language acquisition: The developmental process of learning a new language, encompassing both comprehension and production as well as an understanding of the language's components (e.g., its grammar, phonetics, semantics, etc).
Associative learning: The gradual process of forming links between items based on the statistical regularities of their co-occurrences across developmental time. For example, learning to associate a word with an object based on the regularity of that object being present when that word is used (for a review see, McMurray et al., 2012).
Ostensive naming: Naming something in a highly obvious manner to maximize the likelihood that the listener understands to what the name refers. For example, pointing to something as it is being named.
Competitors: Items that compete for one's attention; such non-targets may draw attention away from a target. Competitor typically refers to a distractor or foil object present on an experimental word learning trial.
Referent selection: Determining the referent of a word, such as an object, an object property (e.g., color) or an object part (e.g., handle). Referent selection frequently refers to choosing the referent from an array of multiple candidates, including a target and at least one competitor. Like fast mapping, referent selection is not to be confused with full word learning (Horst and Samuelson, 2008).
